# Co-Sputtering Crystal Lattice Selection for Rare Earth Metal-Based Multi Cation and Mixed Anion Photochromic Films

**DOI:** 10.3390/nano13040684

**Published:** 2023-02-09

**Authors:** Ming Li, Zewei Shao, Zhongshao Li, Dandan Zhu, Junwei Wang, Smagul Zh. Karazhanov, Ping Jin, Xun Cao

**Affiliations:** 1 State Key Laboratory of High Performance Ceramics and Superfine Microstructure, Shanghai Institute of Ceramics, Chinese Academy of Sciences, Shanghai 200050, China; 2 Center of Materials Science and Optoelectronics Engineering, University of Chinese Academy of Sciences, Beijing 100049, China; 3 Hangzhou Global Scientific and Technological Innovation Center, Zhejiang University, Hangzhou 311215, China; 4 Innovation Cooperation Center (Bangkok), Chinese Academy of Sciences, Shanghai 200040, China; 5 Department for Solar Energy Materials and Technologies, Institute for Energy Technology, 2027 Kjeller, Norway

**Keywords:** co-doping rare-earth oxyhydride [Gd_1−z_Y_z_O_x_H_y_], multi-anion and multi-cation materials, photochromism, lattice selection

## Abstract

Rare-earth oxyhydride (ReO_x_H_y_) films are novel inorganic photochromic materials that have strong potential for applications in windows and optical sensors. Cations greatly influence many material properties and play an important role in the photochromic performance of ReO_x_H_y_. Here we propose a strategy for obtaining Gd_1−z_Y_z_O_x_H_y_ films (z = 1, 0.7, 0.5, 0.4, 0.35, 0.25, 0.15, 0) using one-step direct-current (DC) magnetron co-sputtering. Distinct from the mixed anion systems, such material would belong to the class of mixed anion and mixed cation materials. For Gd_1−z_Y_z_O_x_H_y_ films, different co-doping ratios can help tune the contrast ratio (that is, the difference between coloration and bleaching transmittance) and cycling degradation, which may be related to the lattice constant. X-ray diffraction (XRD) patterns show that the lattice constant increases from 5.38 Å for YO_x_H_y_ to 5.51 Å, corresponding to Gd_0.75_Y_0.25_O_x_H_y_. The contrast ratio, in particular, can be enhanced to 37% from 6.3% by increasing the lattice constant, directly controlled by the co-sputtering power. When the lattice constant decreases, the surface morphology of the sample with the smallest lattice constant is essentially unchanged by testing in air with normal oxidation for 100 days, suggesting great improvement in environment durability. However, the crystal structure cannot be overly compressed, and co-sputtering with Cr gives black opaque films without photochromic properties. Moreover, because the atomic mass of different rare earth elements is different, the critical pressure p* (films deposited at *p* < p* remain metallic dihydrides) is different, and the preparation window is enlarged. Our work provides insights into innovative photochromic materials that can help to achieve commercial production and application.

## 1. Introduction

Photochromic materials can change color and electrical properties when exposed to specific wavelengths of light, which is a reversible process [1]. They have a wide range of applications in many fields, such as optical transistors, smart windows, optoelectronic sensors, optical storage, etc. [2,3,4,5]. Commonly used photochromic materials can be divided into organic and inorganic materials. Most photochromic materials are organic and exhibit narrow spectral absorption bands together with fast switching behavior. However, due to limited stability vs. oxygen, humidity, and heat, as well as ultra-violet (UV) irradiation-induced fatigue, complex processing is often required to tailor the photochromic response and enhance the product’s lifetime. In practical applications, considering the long-term strong light irradiation service weathering resistance and the stability of the preparation process, inorganic photochromic materials are considered to be more promising in this field and have become a new research hotspot.

As a new type of inorganic photochromic material, rare-earth oxyhydride (ReO_x_H_y_) films, based on their effective and reversible optical change characteristics and stable and repeatable preparation technique, have demonstrated tremendous potential for application. Using magnetron sputtering, Mongstad et al. 2011 prepared yttrium oxyhydride films. These films demonstrated noticeably reduced transmittance and resistance when exposed to UV lamp irradiation [6]. There has been a great effort in recent times to study the structural [7], compositional [8], surface [9], electrical [10], and optical properties [11,12] of ReO_x_H_y_ films. Nafezarefi et al. studied the lanthanide Gd, Dy, and Er and found that they had similar photochromic properties to yttrium oxyhydride films. While these ReO_x_H_y_ films have similar cell structures, there are significant differences between the optical contrast and photochromic kinetics [13]. Colombi et al. selected Sc, Y, and Gd, which have a large difference in ionic radius, for their study and found that as the cation radius increased, their lattice constants became larger, and the photochromic contrast was more pronounced, which may be due to the larger lattice reducing the activation barriers for site hopping [14].

To analyze the role of anions in the films, Moldarev made a quantitative analysis of the relationship between the composition of the films and the photochromic properties using TOF-E ERDA, showing that oxidation causes hydrogen to be released [15]. Moreover, as the O/H ratio increases, the photochromic properties decrease, and the bleaching rate increases [10]. To precisely control the O/H ratio and achieve controlled doping of oxygen, Montero successively sputtered YO_x_H_y_ films and Al films used as top protection [16]. Other studies have also mostly used post-oxidation methods to prepare ReO_x_H_y_ films. However, due to the limitations of film thickness and surface porosity, there will be some differences between the surface and internal composition of the samples, which affect the overall photochromic properties. This problem can be effectively avoided by directly preparing transparent ReO_x_H_y_ films through the rational adjustment of argon and hydrogen ratios to achieve precise adjustment of O and H contents.

The suggestions for the chemical formula for the ReO_x_H_y_ are still contradictory: Pishtshev suggested the formula YH_3−2x_O_x_ for yttrium oxyhydride [17]. Colombi has been extended for rare earth metal oxyhydrides as ReO_x_H_3−2x_ [14]. Based on neutron diffraction and synchrotron analysis, the yttrium oxyhydride films are suggested to fit best to YH_2−x_O_x_ [18].

Since oxygen is much more electronegative than hydrogen, with a dissociation energy of 7.4 eV for Y–O bonds and 3.5 eV for Y–H bonds [17], the process of oxygen doping into the lattice is unstoppable. As Pishtshev showed by calculations that as the O/H ratio continues to increase, the enthalpy of YO_x_H_y_ increases, thermodynamic stability decreases, and photochromic properties are lost [19]. Chaykina. et al. enhanced the stability of NdO_x_H_y_ by adding an alumina protective layer via ALD (atomic layer deposition), but the surface encapsulation layer enhanced the bleaching kinetics of the films [20]. Lu et al. improved the generation energy of nanocrystals by doping Sr into CsPbI_3_ nanocrystals, replacing Pb to cause a slight lattice shrinkage, and the environmental stability was enhanced [21,22,23]. These give us an insight into the stability enhancement and advancing optical functionality of the oxyhydride films by partially substituting the cation with another one. The lattice constant can thus be adjusted, which might provide the key to improving the environmental stability of the films.

In the present study, we have attempted to improve the properties of ReO_x_H_y_ films by modulating the lattice constant through rare earth cation alloying. Using Gd as the object of study, we prepare Re(Gd, Y) O_x_H_y_ (the stoichiometric ratio is Gd_1−z_Y_z_O_x_H_y_) thin films directly in one step by reactive magnetron sputtering without the need for a post-oxidation process. The trace oxygen is possibly from the gas sources, even though the nominal purity is 99.99%. Furthermore, we find that the photochromic properties of the films improve as the lattice constant increases, and the oxidation resistance improves as the lattice constant decreases while expanding the preparation window and improving the preparation stability. However, the lattice shrinkage has certain limitations, and when the Cr atoms are doped and the lattice is overly compressed, the band gap of the film is further reduced, and the photochromic properties are lost.

## 2. Materials and Methods

Gd_1−z_Y_z_O_x_H_y_ films were prepared on a quartz substrate with a direct current (DC) magnetron sputtering. Gd (purity 99.9%, 2-in. diameter), Y (purity 99.9%, 2-in. diameter), Ti (purity 99.9%, 2-in. diameter), Cr (purity 99.9%, 2-in. diameter) metal targets were set in the deposition chamber. The Gd_1−z_Y_z_O_x_H_y_ films were deposited using a DC magnetron sputtering at room temperature (RT) in an atmosphere of Ar (purity 4 N) and H_2_ (4 N). The target discharge powers were varied in the range of P_Gd_ = (0–120) W and P_Y_ = (0–100) W ratio to maintain a constant total flux of metal atoms while changing the Y content in the films from z = 0 to 1. The Y/(Gd + Y) fraction, z, was calculated based on the Gd and Y discharge power. X-ray photoelectron spectra (XPS) were implemented to determine the actual ratio of Y doping. For the samples with Y/(Gd + Y) ratios of 0.15, 0.25, 0.35, 0.4, 0.5, and 0.7 used in the synthesis, the actual atomic ratios of Gd/Y in the films were estimated to be 0.18, 0.28, 0.33, 0.41, 0.46, and 0.67, respectively (Figure S1). The power and content of Cr and Ti were derived from the Y-atomic ratio. The base pressure of the deposition chamber was less than 5.0 × 10^−4^ Pa, and the working pressure during deposition was maintained at 0.46 Pa by controlling the mass flow of Ar/H_2_ (4:1). Ar and H_2_ are fed directly, the oxygen we believe comes from an impure argon source or chamber. By adjusting the deposition time, the thickness of all films was around 300 nm, which was determined using an SEM cross-section.

The optical transmittance spectra were measured at 350–2600 nm using an optical spectrophotometer (U-4100, Hitachi High Technologies, Tokyo, Japan). Illumination was performed with a Xe lamp (PLS-SXE 300(UV), PerfectLight, 50 W). Phase identification of films deposited on the quartz glass was performed using X-ray diffraction conducted on a Rigaku Ultima IV diffractometer with Cu Kα radiation (λ = 1.5418 Å) using a 2θ scanning model. The morphology and thickness of the films were measured with a scanning electron microscope (SEM, Hitachi SU8220, Tokyo, Japan) and an atomic force microscope (AFM, SII Nano Technology Ltd., Nanonavi P, Shanghai, China). XPS (ESCA Lab 250, Thermo Fisher Scientific Co. Inc., Waltham, MA, USA) was used to analyze the chemical states of the film. All binding energies were referenced to the C 1s peak (284.8 eV) of adventitious carbon.

## 3. Results

The ReO_x_H_y_ films can respond to different light intensities and change the transmittance of different magnitudes. Physical photographs of the samples before and after the response are shown in Figure S2c. The initial state is yellow and transparent due to inter-band excitation, while changes in optical density occur during the course of illumination, and no color change occurs [6]. The crystal structure of the ReO_x_H_y_ film is that the cations form an fcc skeleton, with the H ions exhibiting –1 valence and the O ions being −2 valence, occupying tetrahedral and octahedral positions in a disordered manner, and the mechanism of photochromism is thought to be related to anion migration [24]. Using NMR, previous studies have demonstrated that the signal of the class of H ions with the highest mobility disappears under illumination, and Shao et al. have proposed a conjecture of “dihydrogen” through the real-time time-dependent density functional theory (RT-TDDFT) method [25,26]. We present a schematic diagram of the photochromic mechanism of ReO_x_H_y_ in Figure S2b, in which two hydrogen ions at different locations are excited using photon energy to form dihydrogen defects [25], leading to a decrease in the transmittance of the film.

Oxidation processes inevitably occur when the films are left in air. In the report, it is mentioned that for YO_x_H_y_ film, the stoichiometric ratio of the sample with better photochromic properties is Y_4_H_10_O, and in the schematic, we also refer to this stoichiometry [19]. When the sample is prepared directly for storage in air, oxygen will continue to enter the lattice, displacing hydrogen into tetrahedral positions, and some of the hydrogen ions will move to octahedral positions or be released. Further, the light will aid this process. This can be found in the X-ray diffraction patterns in Figure S2a, where the strongest diffraction peak of the film (200) is shifted towards a lower angle as the number of illuminations increases, and lattice expansion occurs, indicating the doping of oxygen.

**Figure 1:**
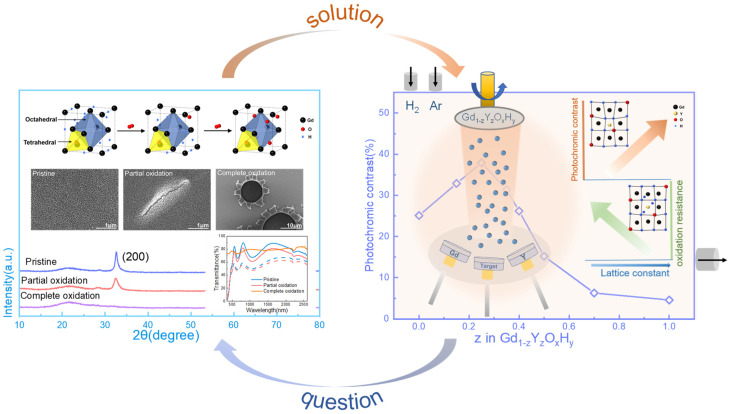
The transmittance spectra, X-ray diffraction patterns, SEM images, and schematic diagrams of lattice structure changes show the oxidation process of thin films. Also shown are co-sputtering film preparation methods, oxidation resistance, and optical contrast around the lattice constant.

Furthermore, as the tetrahedral positions continue to be occupied, the photochromic properties decrease. Eventually, the film becomes an amorphous oxide. This is also seen in the spectrum and XRD patterns of Figure 1, where the maximum optical contrast decreases as the partial oxidation occurs and the intensity of the diffraction peaks of the film gradually decreases, and eventually, the diffraction peaks disappear. The SEM images in Figure 1 show how the film’s surface changes after oxidation. We can see the degree of oxidation from the change in the film’s morphology. The method is shown in Figure 1, right. By adjusting the argon and hydrogen atmosphere, the Re(Gd, Y)O_x_H_y_ film is prepared in one step, and the sputtering power of Y and Gd is adjusted to achieve a gradient of Y and Gd content to achieve different lattice constants.

**Figure 2:**
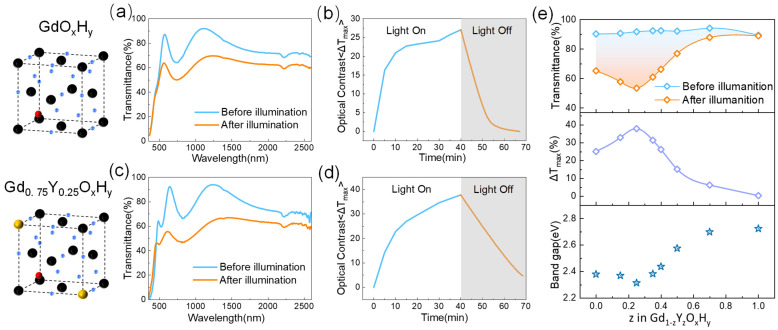
(**a**) The transmittance spectra before and after illumination and (**b**) time-based photodarkening and bleaching curves of GdO_x_H_y_ films. (**c**) The transmittance spectra and (**d**) time-based photodarkening and bleaching curves of Gd_0.75_Y_0.25_O_x_H_y_ films. (**e**) The transmittance spectra, optical contrast, and optical band gap of different Y and Gd sputtering ratios.

Figure 2 shows the cell structure spectra, time-based photodarkening, and bleaching curves of the GdO_x_H_y_ and Gd_0.75_Y_0.25_O_x_H_y_ obtained by co-sputtering. The EDS elemental mapping images in Figure S3 show that the co-sputtering prepared a single phase of uniform composition. We define the maximum optical contrast ΔT as the initial transmittance minus the transmittance after illumination, ΔT = T_0_ − T_t_. For the GdO_x_H_y_ films, the transmittance curves before and after illumination are shown in Figure 2a. After illumination, a significant decrease in transmittance occurs from the visible to the NIR band, with a maximum decrease of up to 27%.

As can be seen in Figure 2b, at 10 min of light, ΔT has reached 70% of the maximum rate of change, and the transmittance quickly returns to its initial state after cessation of illumination. Figure 2d shows the transmittance spectra of Gd_0.75_Y_0.25_O_x_H_y_ with 25% Y content after 30 min of light exposure, has a maximum optical contrast improvement of 37.1% (ΔT ≈ 37%), and has good response performance, returning over 80% within half an hour. Figure S4 shows the maximum optical contrast and the corresponding wavelength transmittance changes after nine cycles for the Gd_0.75_Y_0.25_O_x_H_y_. Although the initial rate of change in the Gd_0.75_Y_0.25_O_x_H_y_ is large, the photochromic properties of the films show a decreasing trend as the number of cycles increases.

Figure 2e shows the photochromic properties of the films with different Gd and Y contents. In the first row of Figure 2e, we can see that the maximum transmittance of the films remains essentially constant as the amount of Y sputtered increases, and the color of the films changes somewhat, as can be seen in Figure S5a, corresponding to the position of the absorption edge on the spectrum that gradually shifts with increasing amounts of Y sputtered. In the second row of Figure 2e, we give the maximum optical contrast data taken from the wavelength with the highest photochromic contrast for the amount of Y sputtered, showing the best optical properties when the amount of Y sputtered is 25%, with the photochromic properties gradually decreasing as the Y content continues to increase. In the chemical formula YH_2-x_O_x_ proposed by Moldarev, yttrium oxyhydride exhibits photochromic behavior in the range 0.45 < δ < 1.5. The strength of the photochromic response is found to decrease with increasing oxygen concentration in the film [8]. The poor photochromic performance of YO_x_H_y_ at a preparation pressure of Ar/H_2_ (40:10) is probably due to the increased oxygen content in the sample at the higher pressure during the preparation process.

**Figure 3:**
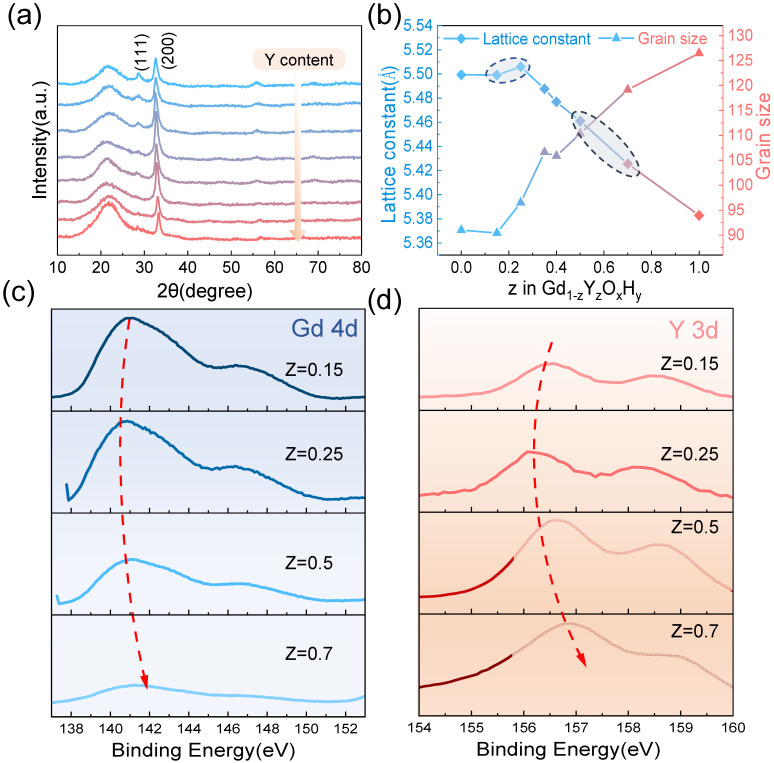
(**a**) The X-ray diffraction pattern and (**b**) the lattice constants and grain size of different Y and Gd content. The XPS spectra show (**c**) Gd 4d and (**d**) Y 3d binding energies.

The XRD patterns In Figure 3a show that at a sputtering pressure of 0.46 Pa, co-doping of Y gradually causes the (111) lattice plane to disappear and the intensity of the (200) lattice plane to decrease gradually. This change in peak level may be indicative of a progressively changing meritocratic orientation of the film, which has been mentioned in previous studies [27]. Figure 3b shows that the lattice constant tends to increase and then decrease as the amount of Y content increases when the lattice constant is maximum, corresponding to the sample with the best optical contrast. Dam et al. compared three rare earth elements with different ionic radii, Gd, Y, and Sc, and found that the larger the ionic radius, the higher the photochromic contrast of the sample, presumably due to the larger lattice helping the anion leap. This interesting phenomenon is also found in our experiments, where the ionic radius of Gd^3+^ is 93.5 pm and Y^3+^ 90 pm. Thus, the tendency for the lattice constant to increase at a lower Y content may be explained by the small difference in the lattice constants of 5.43 Å and 5.32 Å near the critical pressure due to the co-doping of Y reducing the formation energy of the interstitial hydrogen. Moreover, lattice expansion is due to increased hydrogen ions in the interstitial space. We also observe a minimum from the band gap, which laterally accounts for the increase in hydrogen. Tian et al. [28] doped SnS_2_ with Ag and Cu of different ionic radii and found that after doping, significant cell expansion was produced, and the number of reactive sites increased, resulting in improved piezoelectric properties. In addition to lattice expansion favoring ion leap when the lattice constant is increased, more interstitial hydrogen atoms are doped into the cell during preparation, and the increased number of ions involved in the reaction may also be a reason for the better photochromic properties. The grain size graph in Figure 3b shows a trend where the grain size increases as the Y content increases. During the growth of the film, an island growth pattern is generally adopted, and as the deposition time increases, these first formed islands continue to receive new deposited atoms, gradually forming a continuous film, allowing the surface energy to be minimized. For polycrystalline films, the free energy is reduced by reducing the grain boundary area. This creates an intrinsic drive to reduce the grain boundary area with grain growth, which in turn reduces defects at the grain boundaries [29]. Hans et al. have observed the enrichment of oxygen at the grain boundaries with large residual stresses [30]. Thus, the increase in Y content leads to grain growth and reduces grain boundaries to reduce the pathway of oxygen incorporation and enhance the oxidation resistance. This phenomenon may be due to the different deposited atomic mobility of Y and Gd. Additionally, the film growth is affected by substrate, defects, etc., resulting in a slight reduction in grain size for samples with a Y content of 0.15.

We analyze the XPS spectra of Y3d and Gd4d with different Y content (the four samples circled in Figure 3). From top to bottom, the Y content gradually increases. It can be observed that the binding energy tends to decrease and then increase, which corresponds to an increase and then decrease in the lattice constant. The sample with the largest lattice constant in the second row shows a decrease in the Gd4d binding energy compared to the sample with a smaller lattice constant, and the difference in the Y3d binding energy is even more pronounced. This may be due to the lattice contraction caused by the introduction of smaller ionic radius elements by co-doping, resulting in a slight increase in the binding energy of Y and Gd, which contributes to the lattice stability [22,31].

**Figure 4:**
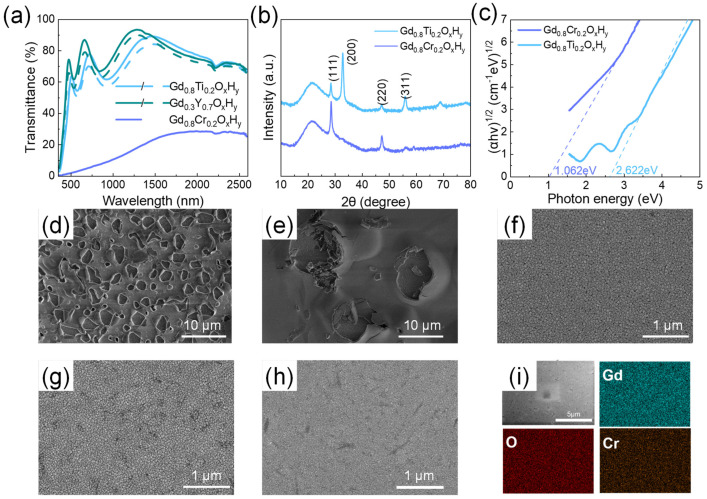
(**a**) The transmittance spectra of Gd_0.3_Y_0.7_O_x_H_y_, Gd_0.8_Ti_0.2_O_x_H_y_, and Gd_0.8_Cr_0.2_O_x_H_y_ films. (**b**) The X-ray diffraction patterns and (**c**) Tauc-plots of Gd_0.8_Ti_0.2_O_x_H_y_ and Gd_0.8_Cr_0.2_O_x_H_y_ films. The surface SEM images of (**d**) GdO_x_H_y_, (**e**) Gd_0.75_Y_0.25_O_x_H_y_, (**f**) Gd0.3Y0.7OxHy, (**g**) Gd_0.8_Ti_0.2_O_x_H_y_, and (**h**) Gd_0.8_Cr_0.2_O_x_H_y_ films placed in air for 100 days; (**i**) The EDS elemental mapping images of the surfaces of the Gd_0.8_Cr_0.2_O_x_H_y_ films.

The samples with the highest and lowest lattice constant are selected for the oxidation experiments. Figure 4d–f shows the surface morphology of the GdO_x_H_y_, Gd_0.75_Y_0.25_O_x_H_y_, and Gd_0.3_Y_0.7_O_x_H_y_ films after 100 days in air. The surface morphology of the Gd_0.3_Y_0.7_O_x_H_y_ is found to be very good after 100 days, with no film deterioration and high oxidation resistance, while the GdO_x_H_y_ and Gd_0.75_Y_0.25_O_x_H_y_ films have severe surface deterioration and reduced densities over the same period. It indicates that the lattice shrinkage may have raised the potential barrier for oxygen doping and slowed down the oxygen doping process. Figure 4a shows the photochromic properties of Gd_0.3_Y_0.7_O_x_H_y_ films, with small variations, where the enhanced oxidation resistance is at the expense of photochromic properties.

We also added the Ti and Cr; the ionic radius of each element is shown in Table 1. The spectra of Gd_0.8_Ti_0.2_O_x_H_y_ and Gd_0.8_Cr_0.2_O_x_H_y_ are shown in Figure 4a; the transmittance of Gd_0.8_Ti_0.2_O_x_H_y_ in both visible and near-infrared regions is above 60%, with a small optical contrast. However, when Cr is doped to form the Gd_0.8_Cr_0.2_O_x_H_y_, the transmittance decreases significantly. Surface roughness calculations yielded 2.8 nm for the Gd_0.8_Cr_0.2_O_x_H_y_. The SEM images of its oxidation in air for 100 days are shown in Figure 4g,h. There is no significant surface deterioration. These suggest that reducing the lattice constant has a role in reducing the oxidation of the sample, but there are limits to the choice of shrinkage lattice parameters. The XRD patterns in Figure 4b show a significant increase in the intensity of the (111) lattice plane and the disappearance of the (200) lattice plane, which is more similar to the XRD patterns of GdH_2_. The EDS elemental mapping images in Figure 4i show that the films obtained with sputtering are homogeneous as a single phase, excluding the local presence of hydrides. The optical band gap in Figure 4c shows a small band gap for the Gd_0.8_Cr_0.2_O_x_H_y_, which results in a transmittance of less than 20% in both the visible and NIR bands. This indicates that when the lattice shrinks excessively, the formation energy barrier for oxygen doping into the lattice is too high to form transparent ReO_x_H_y_ films.

**Figure 5:**
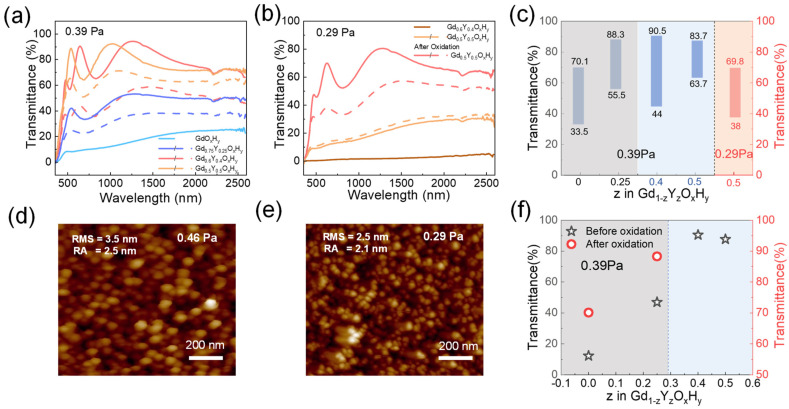
(**a**) The transmittance spectra at 0.39 Pa and (**b**) at 0.29 Pa, (**c**) maximum transmittance and optical contrast at different pressures, and (**f**) maximum transmittance at 0.39 Pa of GdO_x_H_y_, Gd_0.75_Y_0.25_O_x_H_y_, Gd_0.6_Y_0.4_O_x_H_y_, and Gd_0.5_Y_0.5_O_x_H_y_ films. AFM topography images scanned over 1 μm × 1 μm^2^ surface area for Gd_0.5_Y_0.5_O_x_H_y_ film prepared at (**d**) 0.46 Pa and (**e**) 0.29 Pa.

Figure 5 shows the critical preparation pressures p* (films deposited at *p* < p* remain metallic dihydrides) for samples with different co-doping parameters [13]. At a preparation pressure of 0.39 Pa, the Gd_0.75_Y_0.25_O_x_H_y_ and GdO_x_H_y_ films have a low initial state transmittance, but Gd_0.75_Y_0.25_O_x_H_y_ films can respond to light with a decrease in transmittance of about 16.9%. The Gd_0.6_Y_0.4_O_x_H_y_ and Gd_0.5_Y_0.5_O_x_H_y_ can be directly prepared in one step with yellow transparent oxygen-containing hydride films with optical contrast of 46% and 20%, respectively. These films are exposed to the same light hours and conditions. Figure S7 shows the transmittance spectra of GdO_x_H_y_, and Gd_0.75_Y_0.25_O_x_H_y_ films after oxidation. The transmittance of Gd_0.75_Y_0.25_O_x_H_y_ films is higher than that of GdO_x_H_y_ films. Figure 5b shows the spectra of the Gd_0.6_Y_0.4_O_x_H_y_ and Gd_0.5_Y_0.5_O_x_H_y_ films when the pressure is reduced to 0.29 Pa. The Gd_0.6_Y_0.4_O_x_H_y_ has a basic transmittance of 0, while the Gd_0.5_Y_0.5_O_x_H_y_, after oxidation in air, can achieve a transmittance of around 70% and an optical contrast of 31.8%. Figure 5c lists the transmittance at the relevant pressure, and Figure 5f lists the maximum transmittance of prepared samples at 0.39 Pa. Figure 5d,e gives AFM images of Gd_0.5_Y_0.5_O_x_H_y_ prepared at 0.46 Pa and 0.29 Pa. The RMS and RA values show that the surface roughness of the films prepared at lower pressures is relatively lower. It has also been suggested that higher preparation pressures lead to increased porosity on the surface of the films, aiding the entry of oxygen [15]. So, preparing at lower pressures helps to improve the antioxidant capacity of the films. It can be found that as the Y content increases, the critical pressure decreases due to the elemental dependence of the pressure, which may be attributed to the important contribution of the reverse-reflected Ar neutral particles to the total energy flux of the grown film [32]. A suitable amount of Y doping can effectively reduce the critical preparation pressure, expand the preparation window, achieve direct one-step preparation, reduce the lattice damage caused by oxygen doping during the post-oxidation process, and enhance its antioxidant capacity.

## 4. Conclusions

We prepare Gd_1−z_Y_z_O_x_H_y_ films directly in one step using reactive magnetron sputtering without a post-oxidation process and design to regulate lattice constant varying between 5.38 Å and 5.51 Å. For Gd_1−z_Y_z_O_x_H_y_ films, there is a relationship between their photochromic properties and lattice constants. We can find that as the lattice constant increases, the photochromic properties become better, with the best photochromic performance at 25% Y content (5.51 Å) and an optical contrast of 37% (around 37.1% improvement compared to GdO_x_H_y_ film). This may be due to the fact that the larger lattice facilitates the anion transition, and the added hydrogen ions increase the number of reaction sites to aid in the photochromic reaction. As the lattice constant decreases, the oxidation resistance increases, and the films can be left for more than 100 days by comparing SEM images of films with different samples oxidized in air. This is due to lattice contraction, which weakly enhances the binding energy of Y and Gd. However, the lattice shrinkage has certain limitations, and when doping with Cr atoms and over-compressing the lattice, the band gap of the film is further reduced, the transmittance is lower, and the photochromic properties are lost. At the same time, various rare earth elements have varied critical pressures because of the different reflection probabilities for Ar neutral particles that are caused by their different atomic masses. By tuning the co-doping parameters, the preparation pressure of the Gd_1−z_Y_z_O_x_H_y_ films can be reduced, the preparation window can be expanded, and the preparation stability is enhanced.

In summary, in this work, we have designed lattice constants by alloying rare earth cations to modulate the photochromic properties and stability of the Gd_1−z_Y_z_O_x_H_y_ films, providing an idea for future applications of rare earth oxyhydride films.

## Figures and Tables

**Table 1:** Ionic radius and optical contrast of different elements.

Element	Ionic Radius	ΔT
Gd	93.5 pm	27%
Y_(0.25)_	90 pm	37%
Ti_(0.2)_	76 pm	7.9%
Cr_(0.2)_	69 pm	/

## Data Availability

The data presented in this study are available on request from the corresponding author.

## Supplementary Materials

The following supporting information can be downloaded at: https://www.mdpi.com/article/10.3390/nano13040684/s1, Figure S1: The actual ratio of Y/(Gd + Y) as determined by XPS versus the ratio of Y/(Gd + Y) used in the synthesis; Figure S2: (a) The X-ray diffraction patterns of GdO_x_H_y_ films exposed to 10 and 35 times of light. (b) Schematic diagram of the photochromic mechanism. (c) Sample photos before and after illumination; Figure S3: The EDS elemental mapping images of the surfaces of (a) Gd_0.3_Y_0.7_O_x_H_y_ and (b) Gd_0.75_Y_0.25_O_x_H_y_ films; Figure S4: The maximum optical contrast and the change in transmittance of the corresponding wavelength after 9 cycles of Gd_0.75_Y_0.25_O_x_H_y_ films; Figure S5: (a) The sample pictures, (b) Tauc-plots, and (c) transmittance spectra of Y and Gd films with different ratios; Figure S6: The AFM images of (a) GdO_x_H_y_, (b) Gd_0.75_Y_0.25_O_x_H_y_, (c) Gd_0.3_Y_0.7_O_x_H_y_, (d) Gd_0.8_Ti_0.2_O_x_H_y_, and(e) Gd_0.8_Cr_0.2_O_x_H_y_ films; Figure S7: The transmittance spectra of GdO_x_H_y_ and Gd_0.75_Y_0.25_O_x_H_y_ films after oxidation. Reference [33] was cited in Supplementary Materials.

## References

[1] Ke Y., Chen J., Lin G., Wang S., Zhou Y., Yin J., Lee P.S., Long Y. (2019). Smart Windows: Electro-, Thermo-, Mechano-, Photochromics, and Beyond. Adv. Energy Mater..

[2] Kuroiwa H., Inagaki Y., Mutoh K., Abe J. (2019). On-Demand Control of the Photochromic Properties of Naphthopyrans. Adv. Mater..

[3] Li H., Zhao G., Zhu M., Guo J., Wang C. (2022). Robust Large-Sized Photochromic Photonic Crystal Film for Smart Decoration and Anti-Counterfeiting. ACS Appl. Mater. Interfaces.

[4] Shi W., Xing F., Bai Y.L., Hu M., Zhao Y., Li M.X., Zhu S. (2015). High Sensitivity Viologen for a Facile and Versatile Sensor of Base and Solvent Polarity in Solution and Solid State in Air Atmosphere. ACS Appl. Mater. Interfaces.

[5] Yang Z., Du J., Martin L.I.D.J., Feng A., Cosaert E., Zhao B., Liu W., Van Deun R., Vrielinck H., Poelman D. (2021). Designing Photochromic Materials with Large Luminescence Modulation and Strong Photochromic Efficiency for Dual-Mode Rewritable Optical Storage. Adv. Opt. Mater..

[6] Mongstad T., Platzer-Björkman C., Maehlen J., Mooij L., Pivak Y., Dam B., Marstein E., Hauback B., Karazhanov S.Z. (2011). A new thin film photochromic material: Oxygen-containing yttrium hydride. Sol. Energy Mater. Sol. Cells.

[7] Maehlen J.P., Mongstad T.T., You C.C., Karazhanov S. (2013). Lattice contraction in photochromic yttrium hydride. J. Alloys Compd..

[8] Moldarev D., Moro M.V., You C.C., Baba E.M., Karazhanov S.Z., Wolff M., Primetzhofer D. (2018). Yttrium oxyhydrides for photochromic applications: Correlating composition and optical response. Phys. Rev. Mater..

[9] Mongstad T., Platzer-Björkman C., Mæhlen J.P., Hauback B.C., Karazhanov S.Z., Cousin F. (2012). Surface oxide on thin films of yttrium hydride studied by neutron reflectometry. Appl. Phys. Lett..

[10] You C.C., Mongstad T., Marstein E.S., Karazhanov S.Z. (2019). The dependence of structural, electrical, and optical properties on the composition of photochromic yttrium oxyhydride thin films. Materialia.

[11] You C.C., Moldarev D., Mongstad T., Primetzhofer D., Wolff M., Marstein E.S., Karazhanov S.Z. (2017). Enhanced photochromic response in oxygen-containing yttrium hydride thin films transformed by an oxidation process. Sol. Energy Mater. Sol. Cells.

[12] You C.C., Mongstad T., Maehlen J.P., Karazhanov S. (2014). Engineering of the band gap and optical properties of thin films of yttrium hydride. Appl. Phys. Lett..

[13] Nafezarefi F., Schreuders H., Dam B., Cornelius S. (2017). Photochromism of rare-earth metal-oxy-hydrides. Appl. Phys. Lett..

[14] Colombi G., De Krom T., Chaykina D., Cornelius S., Eijt S.W.H., Dam B. (2021). Influence of Cation (RE = Sc, Y, Gd) and O/H Anion Ratio on the Photochromic Properties of REO x H3-2x Thin Films. ACS Photonics.

[15] Moldarev D., Primetzhofer D., You C.C., Karazhanov S.Z., Montero J., Martinsen F., Mongstad T., Marstein E.S., Wolff M. (2018). Composition of photochromic oxygen-containing yttrium hydride films. Sol. Energy Mater. Sol. Cells.

[16] Montero J., Martinsen F.A., Lelis M., Karazhanov S.Z., Hauback B.C., Marstein E.S. (2018). Preparation of yttrium hydride-based photochromic films by reactive magnetron sputtering. Sol. Energy Mater. Sol. Cells.

[17] Pishtshev A., Karazhanov S.Z. (2014). Role of oxygen in materials properties of yttrium trihydride. Solid State Commun..

[18] Sørby M.H., Martinsen F., Karazhanov S.Z., Hauback B.C., Marstein E.S. (2022). On the Crystal Chemistry of Photochromic Yttrium Oxyhydride. Energies.

[19] Pishtshev A., Strugovshchikov E., Karazhanov S. (2019). Conceptual Design of Yttrium Oxyhydrides: Phase Diagram, Structure, and Properties. Cryst. Growth Des..

[20] Chaykina D., Nafezarefi F., Colombi G., Cornelius S., Bannenberg L.J., Schreuders H., Dam B. (2022). Influence of Crystal Structure, Encapsulation, and Annealing on Photochromism in Nd Oxyhydride Thin Films. J. Phys. Chem. C Nanomater. Interfaces.

[21] Liu S., Chen Y., Zhao Y., Xiang W., Liang X. (2020). Doping and surface passivation improve luminescence intensity and stability of CsPbI3 nanocrystals for LEDs. Mater. Lett..

[22] Lu M., Zhang X., Zhang Y., Guo J., Shen X., Yu W.W., Rogach A.L. (2018). Simultaneous Strontium Doping and Chlorine Surface Passivation Improve Luminescence Intensity and Stability of CsPbI3 Nanocrystals Enabling Efficient Light-Emitting Devices. Adv. Mater..

[23] Sun Y.-R., Zhang X., Wang L.-G., Liu Z.-K., Kang N., Zhou N., You W.-L., Li J., Yu X.-F. (2021). Lattice contraction tailoring in perovskite oxides towards improvement of oxygen electrode catalytic activity. Chem. Eng. J..

[24] Colombi G., Cornelius S., Longo A., Dam B. (2020). Structure Model for Anion-Disordered Photochromic Gadolinium Oxyhydride Thin Films. J. Phys. Chem. C.

[25] Chai J., Shao Z., Wang H., Ming C., Oh W., Ye T., Zhang Y., Cao X., Jin P., Zhang S. (2020). Ultrafast processes in photochromic material YHxOy studied by excited-state density functional theory simulation. Sci. China Mater..

[26] Chandran C.V., Schreuders H., Dam B., Janssen J.W.G., Bart J., Kentgens A.P.M., van Bentum P.J.M. (2014). Solid-State NMR Studies of the Photochromic Effects of Thin Films of Oxygen-Containing Yttrium Hydride. J. Phys. Chem. C.

[27] Baba E.M., Montero J., Moldarev D., Moro M.V., Wolff M., Primetzhofer D., Sartori S., Zayim E., Karazhanov S. (2020). Preferential Orientation of Photochromic Gadolinium Oxyhydride Films. Molecules.

[28] Tian W., Han J., Wan L., Li N., Chen D., Xu Q., Li H., Lu J. (2023). Enhanced piezocatalytic activity in ion-doped SnS2 via lattice distortion engineering for BPA degradation and hydrogen production. Nano Energy.

[29] Kapoor M., Thompson G.B. (2015). Role of atomic migration in nanocrystalline stability: Grain size and thin film stress states. Curr. Opin. Solid State Mater. Sci..

[30] Hans M., Tran T.T., Aðalsteinsson S.M., Moldarev D., Moro M.V., Wolff M., Primetzhofer D. (2020). Photochromic Mechanism and Dual-Phase Formation in Oxygen-Containing Rare-Earth Hydride Thin Films. Adv. Opt. Mater..

[31] Zhang J., Zhang L., Cai P., Xue X., Wang M., Zhang J., Tu G. (2019). Enhancing stability of red perovskite nanocrystals through copper substitution for efficient light-emitting diodes. Nano Energy.

[32] Drüsedau T.P., Bock T., John T.-M., Klabunde F., Eckstein W. (1999). Energy transfer into the growing film during sputter deposition: An investigation by calorimetric measurements and Monte Carlo simulations. J. Vac. Sci. Technol. A Vac. Surf. Film..

[33] Zhang Q., Xie L., Zhu Y., Tao Y., Li R., Xu J., Bao S., Jin P. (2019). Photo-thermochromic properties of oxygen-containing yttrium hydride and tungsten oxide composite films. Sol. Energy Mater. Sol. Cells.

