# Supplementary material for: Co-Sputtering Crystal Lattice Selection for Rare Earth Metal-Based Multi Cation and Mixed Anion Photochromic Films
Source: Nanomaterials (Basel). 2023 Feb 9;13(4):684. doi: 10.3390/nano13040684 (PMC9960944; doi:10.3390/nano13040684)
Supplement: Supplementary file 1 [file nanomaterials-13-00684-s001.zip › nanomaterials-2181364-supplementary.pdf]

nanomaterials

Supplementary Materials

Co-Sputtering Crystal Lattice Selection for Rare Earth Metal-Based Multi Cation and Mixed Anion Photochromic Films

Ming Li [1,2], Zewei Shao [3], Zhongshao Li [1], Dandan Zhu [1,2], Junwei Wang [4], Smagul Zh. Karazhanov [5], Ping Jin [1] and Xun Cao [1,2,*]

1 State Key Laboratory of High Performance Ceramics and Superfine Microstructure, Shanghai Institute of Ceramics, Chinese Academy of Sciences, Shanghai 200050, China
2 Center of Materials Science and Optoelectronics Engineering, University of Chinese Academy of Sciences, Beijing 100049, China
3 Hangzhou Global Scientific and Technological Innovation Center, Zhejiang University, Hangzhou 311215, China
4 Innovation Cooperation Center (Bangkok), Chinese Academy of Sciences, Shanghai 20040, China
5 Department for Solar Energy Materials and Technologies, Institute for Energy Technology, 2027 Kjeller, Norway
* Correspondence: cxun@mail.sic.ac.cn

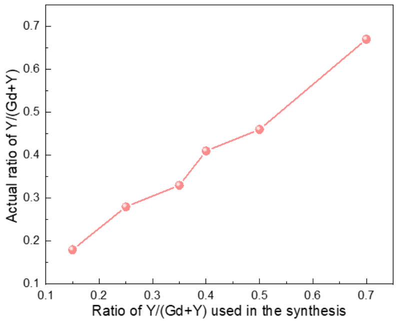

Figure S1. The actual ratio of Y/(Gd+Y) as determined by XPS versus the ratio of Y/(Gd+Y) used in the synthesis.

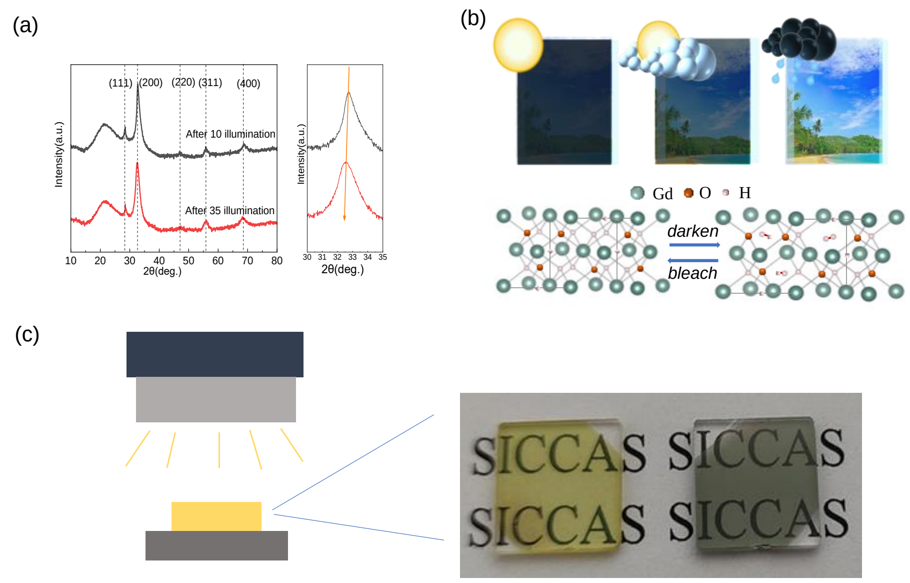

Figure S2. (**a**) The X-ray diffraction patterns of GdO_xH_y films exposed to 10 and 35 times of light. (**b**) Schematic diagram of the photochromic mechanism. (**c**) Sample photos before and after illumination [1, 2].

It is clear from the displacement of the characteristic peaks that the expansion of the lattice occurs with increasing light exposure, which may be due to the entry of oxygen.

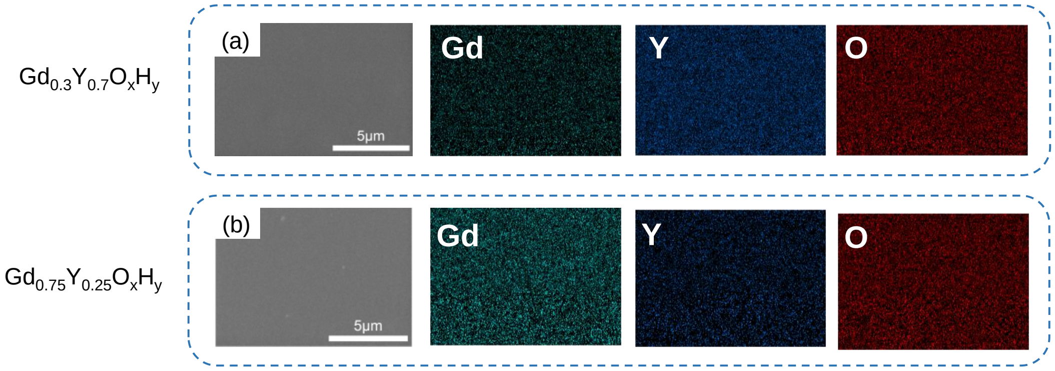

Figure S3. The EDS elemental mapping images of the surfaces of (**a**) $Gd_{0.3}Y_{0.7}O_xH_y$ and (**b**) $Gd_{0.75}Y_{0.25}O_xH_y$ films shows a uniform distribution of the elements on the surface of the films, indicating that a homogeneous phase is formed during the co-sputtering process, rather than a mixture of two substances.

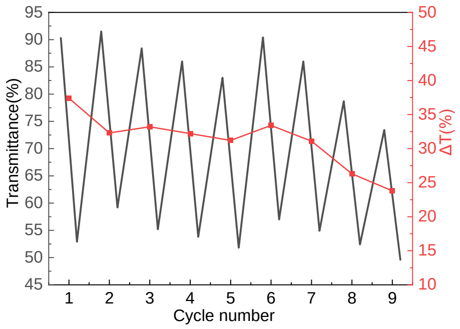

Figure S4. The maximum optical contrast and the change in transmittance of the corresponding wavelength after 9 cycles of $Gd_{0.75}Y_{0.25}O_xH_y$ films. The cycling process was 30 min of light and 30 min of heating at 50°C. It has been previously shown that heating provides energy to help the recovery of the samples[3].

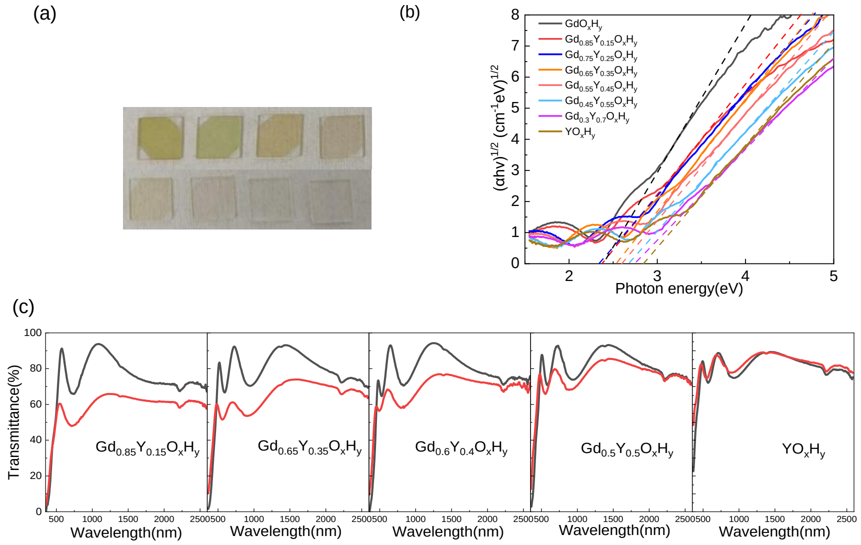

Figure S5. (**a**) The sample pictures, (**b**) Tauc-plots, and (**c**) transmittance spectra of Y and Gd films with different ratios.

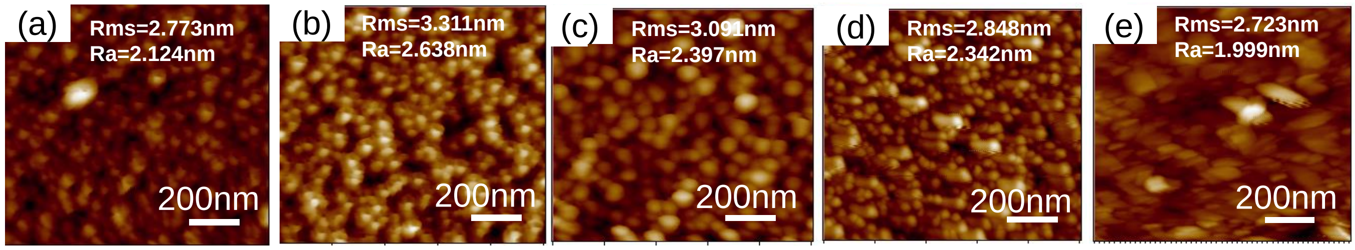

Figure S6. The AFM images of (**a**) GdO_xH_y, (**b**) $Gd_{0.75}Y_{0.25}O_xH_y$, (**c**) $Gd_{0.3}Y_{0.7}O_xH_y$, (**d**) $Gd_{0.8}Ti_{0.2}O_xH_y$, and (**e**) $Gd_{0.8}Cr_{0.2}O_xH_y$ films.

The co-sputtering process inevitably leads to an increase in surface roughness. However, the surface roughness decreases gradually with the decrease of the lattice constant.

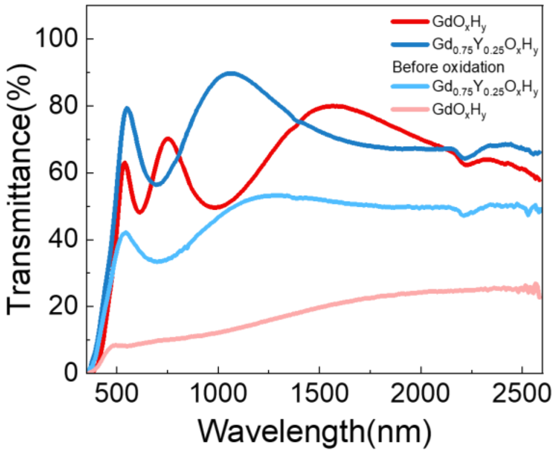

Figure S7. The transmittance spectra of GdO_xH_y and $Gd_{0.75}Y_{0.25}O_xH_y$ films after oxidation.

References

1. Chai, J.; Shao, Z.; Wang, H.; Ming, C.; Oh, W.; Ye, T.; Zhang, Y.; Cao, X.; Jin, P.; Zhang, S.; et al. Ultrafast processes in photochromic material YHxOy studied by excited-state density functional theory simulation. *Sci. China Mater.* **2020**, *63*, 1579–1587. https://doi.org/10.1007/s40843-020-134.
2. Chandran, C.V.; Schreuders, H.; Dam, B.; Janssen, J.W.G.; Bart, J.; Kentgens, A.P.M.; van Bentum, P.J.M. Solid-State NMR Studies of the Photochromic Effects of Thin Films of Oxygen-Containing Yttrium Hydride. *J. Phys. Chem. C* **2014**, *118*, 22935–22942. https://doi.org/10.1021/jp507248c.
3. Zhang, Q.; Xie, L.; Zhu, Y.; Tao, Y.; Li, R.;Xu, J.; Bao S.; Jin P. Photo-thermochromic properties of oxygen-containing yttrium hydride and tungsten oxide composite films. *Sol. Energy Mater. Sol. Cells*. **2019**, *200,* 109930. doi:10.1016/j.solmat.2019.109930.
